# Maternal obesogenic diet induces endometrial hyperplasia, an early hallmark of endometrial cancer, in a diethylstilbestrol mouse model

**DOI:** 10.1371/journal.pone.0186390

**Published:** 2018-05-18

**Authors:** Theresa O. Owuor, Michaela Reid, Lauren Reschke, Ian Hagemann, Suellen Greco, Zeel Modi, Kelle H. Moley

**Affiliations:** 1 Department of Obstetrics and Gynecology, Washington University School of Medicine, St. Louis, MO, United States of America; 2 Department of Pathology and Immunology, Washington University School of Medicine, St. Louis, MO, United States of America; 3 Division of Comparative Medicine, Washington University School of Medicine, St. Louis, MO, United States of America; National Cancer Institute, UNITED STATES

## Abstract

Thirty-eight percent of US adult women are obese, meaning that more children are now born of overweight and obese mothers, leading to an increase in predisposition to several adult onset diseases. To explore this phenomenon, we developed a maternal obesity animal model by feeding mice a diet composed of high fat/ high sugar (HF/HS) and assessed both maternal diet and offspring diet on the development of endometrial cancer (ECa). We show that maternal diet by itself did not lead to ECa initiation in wildtype offspring of the C57Bl/6J mouse strain. While offspring fed a HF/HS post-weaning diet resulted in poor metabolic health and decreased uterine weight (regardless of maternal diet), it did not lead to ECa. We also investigated the effects of the maternal obesogenic diet on ECa development in a Diethylstilbestrol (DES) carcinogenesis mouse model. All mice injected with DES had reproductive tract lesions including decreased number of glands, condensed and hyalinized endometrial stroma, and fibrosis and increased collagen deposition that in some mice extended into the myometrium resulting in extensive disruption and loss of the inner and outer muscular layers. Fifty percent of DES mice that were exposed to maternal HF/HS diet developed several features indicative of the initial stages of carcinogenesis including focal glandular and atypical endometrial hyperplasia versus 0% of their Chow counterparts. There was an increase in phospho-Akt expression in DES mice exposed to maternal HF/HS diet, a regulator of persistent proliferation in the endometrium, and no difference in total Akt, phospho-PTEN and total PTEN expression. In summary, maternal HF/HS diet exposure induces endometrial hyperplasia and other precancerous phenotypes in mice treated with DES. This study suggests that maternal obesity alone is not sufficient for the development of ECa, but has an additive effect in the presence of a secondary insult such as DES.

## Introduction

Endometrial cancer (ECa) is the most common of all gynecological cancers and the fourth most common cancer in women in the United States[1]. Approximately 635,437 women are living with ECa, and 10,470 deaths and 60,050 new cases are expected in 2016[2]. Several factors including exposure to unopposed high estrogen levels, family history, age, radiation therapy, mutations, and obesity, have been linked to an increased risk of ECa[3]. According to the Centers for Disease Control, the prevalence of obesity among women is 38.3%[4]. Several epidemiological studies have associated an increased risk of ECa with weight gain during adulthood[5–7]. For example, Thomas et al. reported that women 45 years or older with a body mass index (BMI) of at least 35 had a greater risk of endometrial cancer (40–56%) than women of the same age with a BMI of 18 to 25 (4–14%). Additionally, adult weight gain was more strongly associated with type I endometrial cancer than type II[5]. The link between ECa and obesity is still unclear, but three possible mechanisms have been proposed: (1) First, obese women have a higher level of circulating estrogen as a result of the peripheral conversion of androgen in adipose tissue to estrogen such as estradiol and estrone[8]. (2) Second, obese women have an increased production of adipokines, namely leptin and adiponectin, from adipose tissue which has been shown to stimulate cancer cells, be involved in angiogenesis, cell apoptosis, cell invasiveness, and create a pro-inflammatory state via production of cytokines such as Interleukins 6 and 12, and Tumor necrosis factor alpha [9]. (3) Third, obesity leads to the development of hyperinsulinemia resulting from chronic insulin resistance, which has been associated with complex molecular changes and interactions that favor tumor formation[10, 11].

While there are several studies linking obesity to endometrial cancer, the link between maternal obesity, a maternal obeseogenic diet, and ECa development in offspring has been less explored. Ninety percent of obese women are within the reproductive ages of 20–39 years,[4] meaning that children born today are more likely to be exposed to an obeseogenic environment both *in utero* and during early childhood. It has been shown that maternal body mass index during pregnancy is positively associated with overall fetal mortality. Exposure to maternal obesity during gestation and lactation not only influence fetal and infant development during sensitive periods, but additionally has been associated with long term health outcomes such as development of cardiovascular disease and type 2 diabetes later in life[12]^,^[13, 14]. Increasing evidence suggests *in utero* exposure leads to early- life programming through environmental, genetic, and epigenetic mechanisms[15]. In several animal models, maternal obesity and maternal HF/HS diet has been shown to lead to metabolic impairments in offspring including obesity, fatty liver disease, insulin resistance, and diabetes[16–19]. Studies have also linked a high fat maternal diet in mice to prostate hyperplasia in male offspring,[20] and to an increase in breast cancer risk in female offspring in rodent models and in women[21, 22]. Sexual dimorphism in response to developmental programming stimuli has been reported across a range of model organisms. Direct comparisons between male and female offspring have been made predominantly in rats[23–27], but sex-specific differences are also reported in mice[28, 29]. Data support that male rat offspring are more susceptible to developmentally programmed hypertension than female offspring in studies where the sexes are directly compared[24, 30–34]. The effect is not entirely consistent, however, as a maternal high-fat diet has been reported to elevate blood pressure in female offspring only [23].

In mammals, reproductive tract development starts during gestation, and in humans, the uterus can be identified as early as 19 weeks gestation[35, 36]. Reproductive tract development continues following birth through the onset of puberty creating a window of susceptibility (*in utero* to puberty) to environmental factors that could potentially influence the development and health of the uterus, possibly leading to increased risk of ECa in adulthood[36]. In 1997, Walker *et al*. found evidence of reproductive tract cancers including endometrial, ovarian, and breast tumors in offspring of female CD-1 mice exposed to a high-fat diet (29% high fat vs. 2.6% low fat from corn oil). Specifically, these mothers were only exposed to a high fat diet prenatally, and were then fed a low fat diet during their gestational period, suggesting that maternal metabolic dysfunction and obesity may have lasting influences on reproductive tract development in offspring[35]. In a follow up study, the authors did not find a significant difference in the frequency of reproductive tract tumors when they perinatally exposed offspring to different types of fat[37]. There was no further follow up studies in regards to the endometrial carcinomas that they observed[35].

Our objective was to develop a mouse model to test the hypothesis that maternal obesity contributes to ECa risk in the offspring. We show that exposure to a maternal HF/HS diet is not by itself sufficient to induce ECa in offspring. We further show that there is no additive adverse effect on uterine health when exposed offspring are then fed the HF/HS diet similar to their mothers. We then introduced diethylstilbestrol (DES) as a secondary insult to maternal diet exposure. DES is a potent estrogen synthetic that induces a wide range of reproductive tract abnormalities and cancers in mice and humans[38],[39]. Our data shows that fifty percent of the offspring exposed to both maternal HF/HS diet and maternal DES injection develop initial phenotypes of ECa such as glandular and endometrial hyperplasia at 24 weeks compared to 0% of their Chow counterparts. In summary, we demonstrate that a second oncogenic insult is required for the initiation of ECa in mice when exposed to a maternal HF/HS diet. Determining the effect of maternal obesity on offspring is fundamental to achieving a better understanding of possible additive risk factors for ECa. *In utero* and early life exposure to a HF/HS diet can influence uterine development and further sensitize the endometrium to secondary insults originating from carcinogens such as DES.

## Methods

### Animal diet and treatment

**Fig 1:**
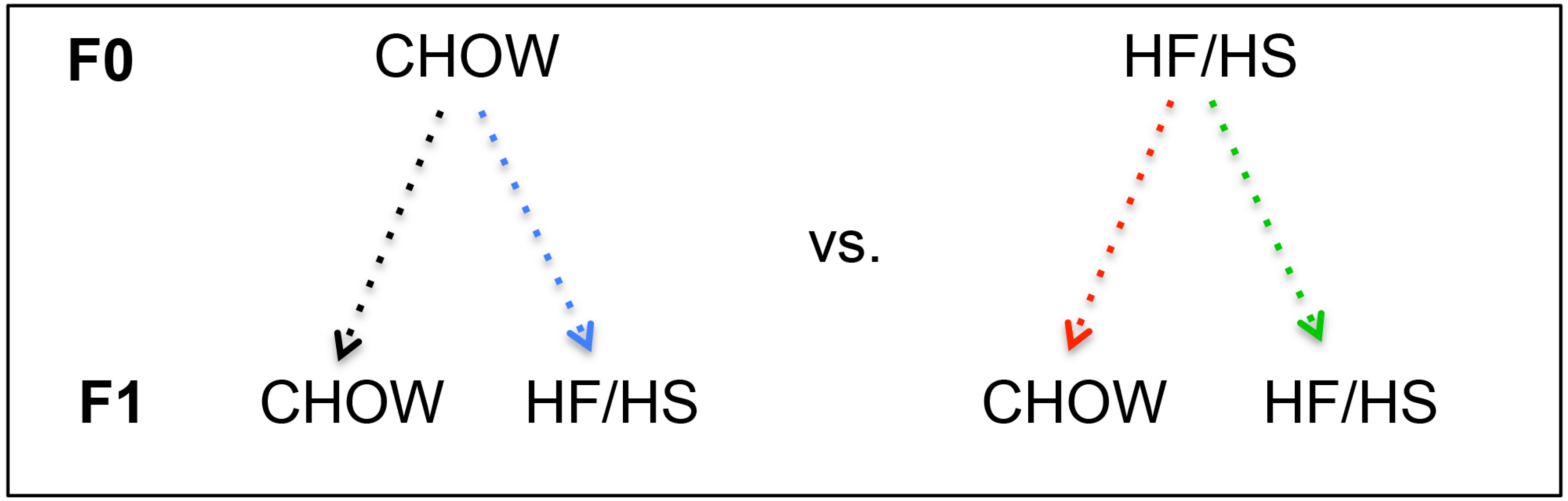
Schematic representation of experimental model. Four-week-old C57Bl/6J female mice fed control (Chow, 13% kcal from fat) or a high-fat high-sugar (HF/HS; 59.4% kcal from fat) diet. After more than 4 weeks of dietary exposure, dams were mated to chow-fed C57Bl/6J studs. All resulting F1 pups were exposed to the mom’s diet in utero and during lactation. We had four cohorts as follow; F0 Chow -F1 Chow, F0 HF/HS -F1 Chow, F0 Chow - F1 HF/HS, F0 HF/HS - F1 HF/HS.

The Animal Studies Committee at Washington University School of Medicine approved animal procedures and protocols. Four-week-old C57Bl/6J female mice were fed a control Chow (PicoLab Rodent Diet 20; 13.2% fat, 62% carbohydrates [3.2% sucrose]) or a HF/HS diet (TestDiet, 58R3; 59.4% fat, 26% carbohydrates [17% sucrose].[6, 20]. After at least four weeks of dietary exposure, dams were mated to Chow-fed C57Bl/6J studs. Resulting offspring were left with their mothers until weaning at postnatal day 21 (P21) and then fed a Chow or HF/HS diet making up 4 cohorts as follows: F0 Chow—F1 Chow, F0 Chow—F1 HFHS, F0 HF/HS—F1 Chow, and F0 HF/HS—F1 HF/HS (Fig 1). The offspring were then sacrificed during the estrous phase at 39 weeks, as the reproductive life of female mice starts to decline around 9 months (~36 weeks) of age. The 72-week mice were more difficult to catch in estrous, however we confirmed that they were menopausal by examining the ovaries by H&E and counting antral follicles. The metabolic profile of these F1 offspring at 39 and 72 weeks were prior to sacrifice. For the DES experiment, the female offspring from both Chow and HF/HS-fed dams were injected with 2 ug/pup/day of DES or corn oil vehicle (Veh)[40] on neonatal days 2, 4 and 6, resulting in 4 cohorts as follows: F0 Chow—Veh, F0 HF/HS—Veh, F0 Chow—DES, F0 HF/HS—DES. Both vehicle and DES mice were fed a Chow diet post-weaning, and then sacrificed at 16 or 24 weeks of age.

### Metabolic analysis

For the glucose tolerance test, 39-week-old mice were fasted for six hours and injected intraperitoneally with 10% glucose at a dose of 1 mg/g of body weight. A glucometer (Contour TS; Bayer) was used to measure glucose from whole blood at 0, 15, 30, 60, 90 and 120-minute intervals. At 72 weeks of age, mice were weighed, and body composition including percent fat was analyzed by quantitative magnetic resonance imaging (EchoMRI- 900). Mice were fasted for 6 hours and fasting triglycerides and cholesterol serum levels were measure by the Infinity Triglyceride and Cholesterol Reagent kits (Fisher Diagnostics). Fasting serum insulin was also measured using the rat/mouse insulin enzyme-linked immunosorbent assay kit (Crystal Chem) as per manufacturer’s instructions.

### Histology

One uterine horn per mouse was fixed overnight in 10% neutral buffered formalin, processed in increasing concentrations of ethanol, and paraffin embedded. Tissues sections (5μm) were processed for histological analysis. Sections were stained with Hematoxylin and Eosin and analyzed by two pathologists blinded to the experimental exposures. For immunohistochemistry, sections were incubated with antibody specific to phospho-histone H3 (pHH3), alpha smooth muscle actin (αSMA) and cytokeratin 8 (CK8) (S1 Table), then with goat HRP-conjugated secondary antibody (Santa Cruz Biotechnology, 1:1000). Slides were developed for 5 minutes with a Vector DAB Kit (Vector Laboratories) per manufacturer's protocol, counterstained in CAT Hematoxylin. Representative images were taken on a Nikon Eclipse E800 upright microscope with an Olympus DP71 camera and manufacturer's software. We used primary antibody against pHH3 for immunofluorescence (S1 Table) followed by goat Alexa Fluor 488—conjugated secondary antibody (Molecular Probes, 1:1000). Sections were counterstained with DAPI imaged by confocal microscopy (Leica TCS SPE/DMI4000B; Leica Application Suite software).

### Immunoblotting

One uterine horn was cut into four pieces, flash-frozen, and processed for immunoblotting or RNA-Sequencing. Sections were lysed in RIPA buffer, and 20 μg of total protein lysate loaded per lane. Immunoblots were incubated at 4°C in TBST/5% BSA with appropriate primary antibodies (S1 Table) followed by goat horseradish peroxidase—conjugated secondary antibody (Santa Cruz Biotechnology, 1:5000). Chemiluminescence was detected using the Super Signal West Pico kit (Thermo Scientific) and quantified using Fiji Image J. software.[41]

### Statistical analyses

Data is expressed as mean ± standard error of the mean (SEM) for normally distributed values. The data is from ≥10 Chow- or HF/HS-exposed and ≥3 in the DES female F1 offspring. All offspring data was calculated using 2-way analysis of variance (ANOVA) followed by Tukey’s multiple comparison’s test except where noted. We used GraphPad PRISM (Version 6.0, La Jolla) for statistical analyses. For all tests, *P* < 0.05 was considered statistically significant.

## Results

### Maternal HF/HS diet exposure leads to metabolic syndrome in mice

**Fig 2:**
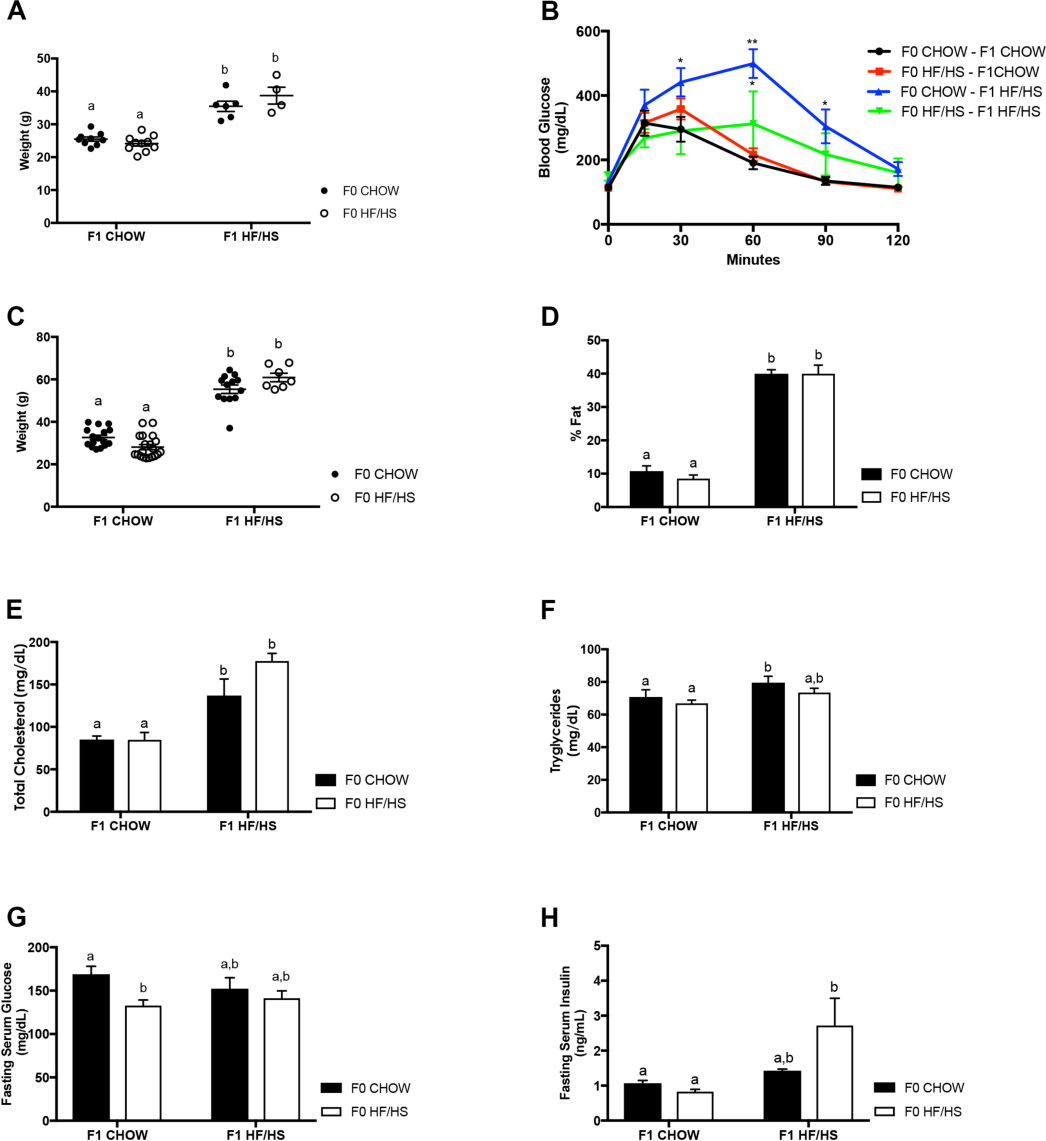
Metabolic consequences of HF/HS exposure in 39 and 72-week-old C57Bl/6J wildtype female offspring. **39-week data of** body weight (**A**) and impaired glucose tolerance (**B**). Body weight (**C**), percent fat (**D**) and cholesterol (**E**) and triglyceride (**F**), fasting serum glucose (**G**) and insulin (**H**) levels at 72 weeks. N > 5 mice per cohort, p<0.05, 2-way ANOVA followed by Tukey’s multiple comparison test. Same letters mean there’s no significant difference between conditions, different letters represent statistically significant differences.

As previously discussed, four-week-old C57Bl/6J female mice were fed control (Chow) or a high-fat high-sugar (HF/HS) diet as previously described. After more than 4 weeks of dietary exposure, these F0 dams were mated to Chow-fed C57Bl/6J studs. All resulting pups were exposed to the mother’s diet until weaning. The F1 offspring were then fed a Chow or a HF/HS post-weaning resulting in four F1 cohorts (Fig 1). We assessed the metabolic profile of these F1 offspring at 39 and 72 weeks before sacrifice. At 39 weeks, F1 mice fed a HF/HS diet were significantly heavier than those on a Chow diet regardless of maternal diet (Fig 2A). F1 Cohorts that were fed a Chow diet showed no significant impairment in glucose tolerance (Fig 2B). Both F1 cohorts that were fed a HF/HS diet were glucose intolerant with the F0 Chow—F1 HF/HS cohort showing a greater impairment than the F0 HF/HS—F1 HF/HS cohort, suggesting that these mice have been reprogrammed in utero to the HF/HS environment and are therefore less disposed to impaired glucose homeostasis (Fig 2B). At 72 weeks, F1 mice fed a HF/HS diet were significantly heavier than those on a Chow diet regardless of their mother’s diet (Fig 2C). F0 HF/HS—F1 HF/HS cohort mice, which were exposed to the HF/HS diet throughout their lifespan, tended to be heavier than the F0 Chow—F1 HF/HS mice, however this difference was not statistically significant (Fig 2C). F1 mice fed a HF/HS diet all had significantly higher percent fat and elevated cholesterol (Fig 2D and 2E). The F0 HF/HS—F1 HF/HS cohort also had slightly higher cholesterol that the F0 Chow—F1 HF/HS, although this was not statistically significant (Fig 2E). In general, triglycerides were elevated in the mice fed a HF/HS diet but were significantly elevated only in the F0 Chow—F1 HF/HS compared to the F0 HF/HS—F1 Chow cohort (Fig 2F). Fasting serum glucose was significantly lower in the F0 HF/HS—F1 Chow group than all other groups (Fig 2G) while mice fed a HF/HS diet post-weaning had higher fasting insulin levels and this was statistically significant in the F0 HF/HS—F1 HF/HS cohort (Fig 2H). In summary, the mice fed a HF/HS diet post-weaning had several features of metabolic syndrome including increased weight gain, impaired glucose tolerance, increased fat, and elevated cholesterol and serum insulin regardless of maternal diet. Exposure to the maternal HF/HS diet did not affect most of these metabolic features in the F1 offspring with the exception of glucose tolerance in the F0 HF/HS—F1 HF/HS cohort.

### Maternal or direct exposure to HF/HS diet does not lead to ECa development in wild-type C57Bl/6J offspring

**Fig 3:**
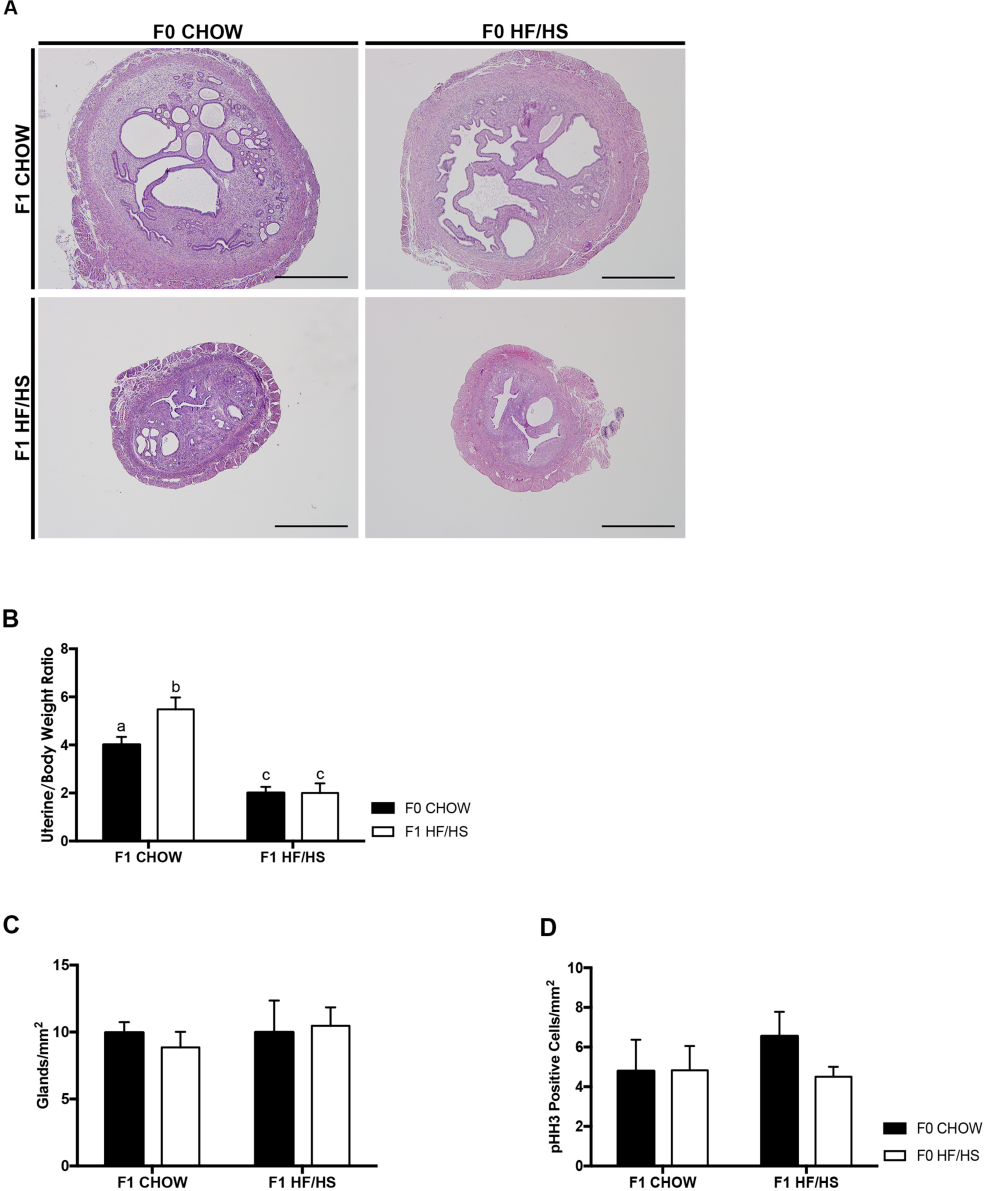
HF/HS diet exposure does not lead to early hallmarks of ECa at 72 weeks. Representative images of H&E staining of uterine slides (**A**). Uterine weight normalized to mouse body weight (**B**). Graphs showing number of glands per stromal area (**C**) and quantification of phospho-HH3 positive cells (**D**). N > 10 mice per cohort, *p<0.05, 2-way ANOVA followed by Tukey’s multiple comparison test. Same letters mean there is no significant difference between conditions, different letters represent statistically significant differences. Scale bars = 1mm at 4X objective.

We performed histological analysis of uteri dissected from these mice. All mice had normal uteri at 39 weeks (data not shown) so were not assessed further. At 72 weeks some of the mice exhibited increased endometrial volume and complexity (2 out of 16 F0 Chow—F1 Chow, and 2 out of 10 F0 HF/HS—F1 Chow) (Fig 3A, S2 Table). The uteri of some mice had cystic dilation of their lumen (2 out of 16 F0 Chow—F1 Chow) and other mice had hyalinized stroma (2 out of 16 F0 Chow—F1 Chow). One mouse in the F0 Chow—F1 Chow cohort exhibited neutrophil infiltration that could be attributed to estrous cycling (S2 Table). Mice fed a HF/HS diet post-weaning had significantly smaller uteri to body weight ratio than those on a Chow diet, regardless of maternal diet (Fig 3B). F0 HF/HS—F1 Chow had significantly heavier uteri than all other groups (Fig 3B). There was no difference in the number of glands in all four groups (Fig 3C). Apart from these phenotypes, all mice had normal uteri with no histological lesions indicative of ECa (S2 Table). We also assessed cell proliferation using phospho-histone H3 (pHH3) staining and saw a trend towards a slight increase in proliferation in uteri of the F0 Chow—F1 HF/HS cohort, but overall there were no significant differences in number of pHH3 positive cells in all cohorts of mice (Fig 3D).

PTEN is a tumor suppressor gene that regulates and inhibits the Akt/PI3K pathway by dephosphorylating Akt, and thus in its absence, this pathway stimulates aberrant growth, proliferation and survival of tumor cells[42]. Post-translational phosphorylation of PTEN at serine 380 (S380) inactivates the protein. We observed an increase in phospho-PTEN expression but noted a decrease in phospho-Akt (S473) in all three cohorts of mice that were exposed to the HF/HS diet relative to the F0 Chow—F1 Chow group. Expression of these proteins varied among mice within the same group. There was no change in expression in the total PTEN, or Akt protein (S1A Fig). This data suggests that any exposure to the HF/HS diet results in a reduction in the activation of the Akt pathway in these mice.

### HF/HS diet exposure does not impact circulating estradiol and estrone levels

**Fig 4:**
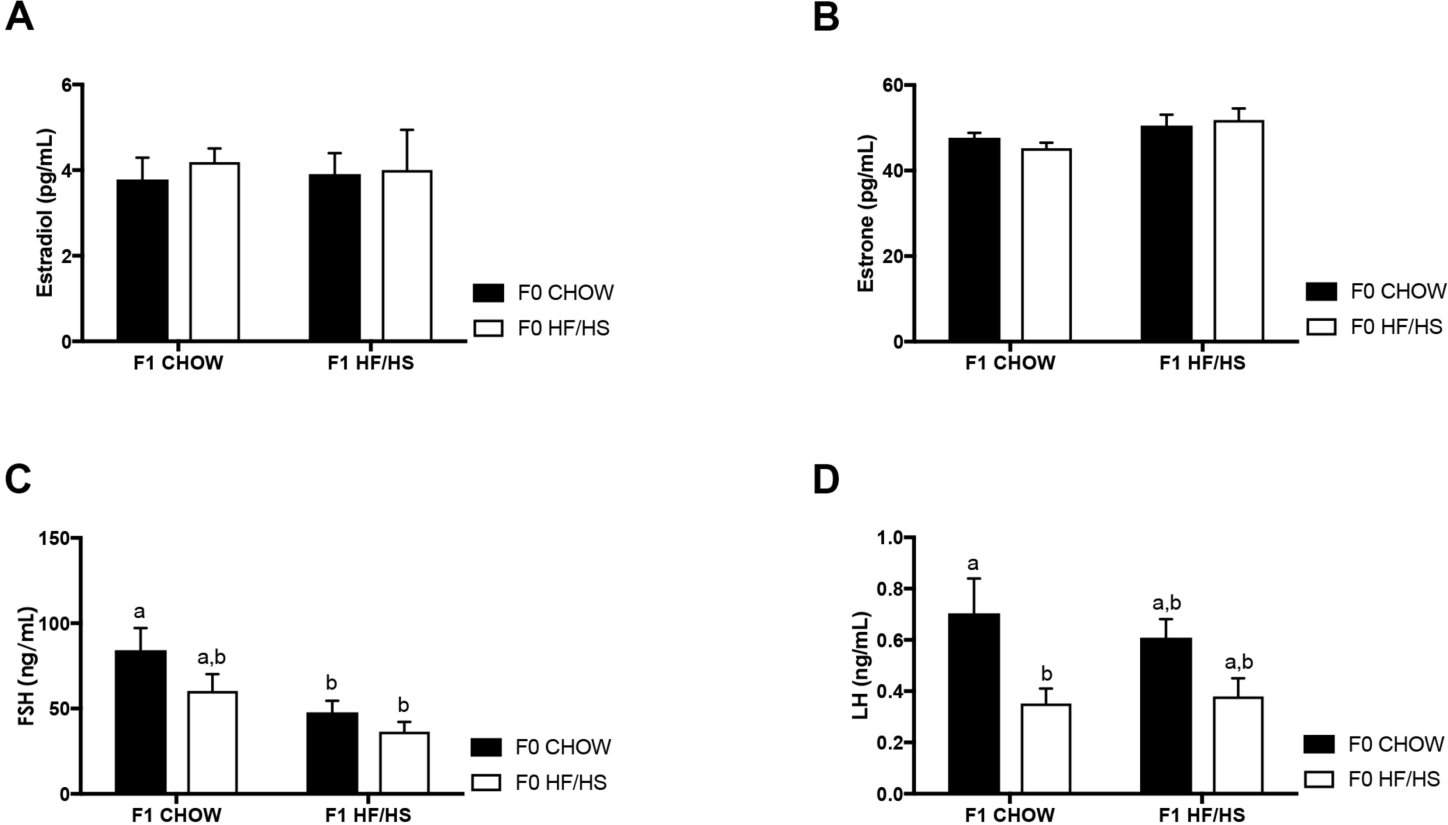
Serum hormone levels in 72 week-old F1 offspring. Estradiol (**A**), Estrone (**B**), FSH (**C**) and LH (**D**). N > 5 mice per cohort, *p<0.05, 2-way ANOVA followed by Tukey’s multiple comparison test. Same letters mean there is no significant difference between conditions, different letters represent statistically significant differences at p<0.01.

After menopause, ovaries cease production of both estrogen and progesterone and adipose tissue becomes the main source of circulating estrogen. In overweight and obese women, high circulating levels of estrogen are explained by an increase in peripheral androgen conversion to estrogen via increased aromatase activity in adipocytes [8]. We next assessed serum hormonal levels in the 72-week-old F1 offspring. There was no difference in levels of estradiol (Fig 4A) or estrone (Fig 4B) in the different cohorts of mice. The level of estrone was slightly but not significantly higher in the mice fed a HF/HS diet. In this study, while the mice were obese, circulating estradiol levels did not change. There was also no change in protein expression of the estrogen receptor α in mice exposed to a HF/HS diet (S1B Fig).

### Maternal diet induces ECa development in DES treated mice

**Fig 5:**
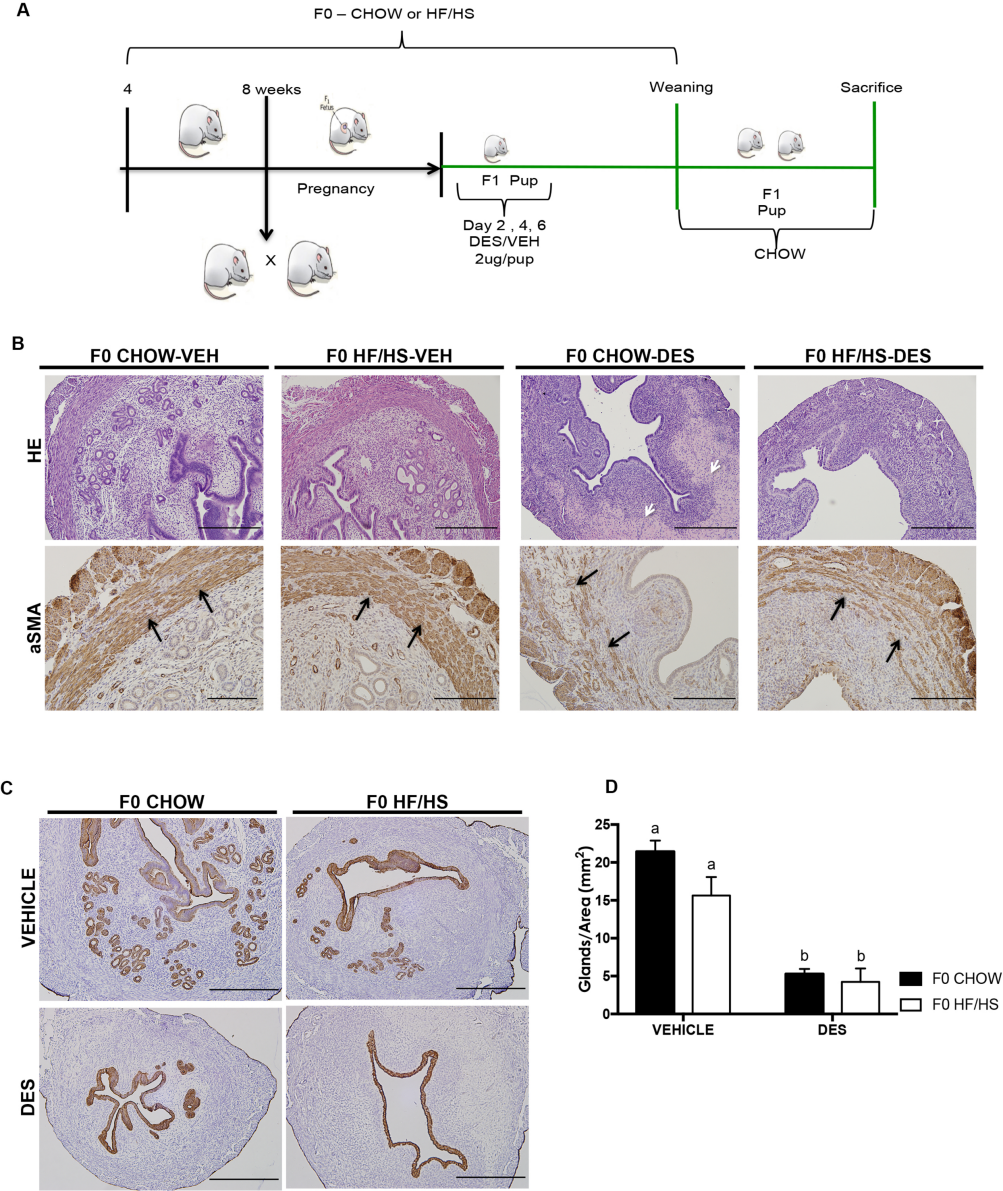
DES exposure induces uterine lesions in C57Bl/6J mice. Schematic representation of DES experimental model. Four-week-old C57Bl/6J female mice fed either control Chow or HF/HS diet for at least 4 weeks then mated to C57Bl/6J males. Female offspring were injected with 2ug/pup/day of DES or vehicle on neonatal days 2, 4 and 6 and fed control chow after weaning until sacrifice (**A**). H&E staining of uterine slides from F0 CHOW-VEH, F0 HF/HS-VEH, F0 CHOW-DES and F0 HF/HS-DES (**B, upper panel**). Representative images of aSMA staining of myometrium and serosa (**B, lower panel**), Scale bars = 500μm at 20X objective. CK8 staining of lumen and glands showing fewer glands in F0 CHOW-DES and F0 HF/HS-DES cohorts (**C**). Quantification of number of glands (**D**). Scale bars = 500 μm at 10X. Same letters mean there is no significant difference between conditions; different letters represent statistically significant differences at p<0.01.

**Fig 6:**
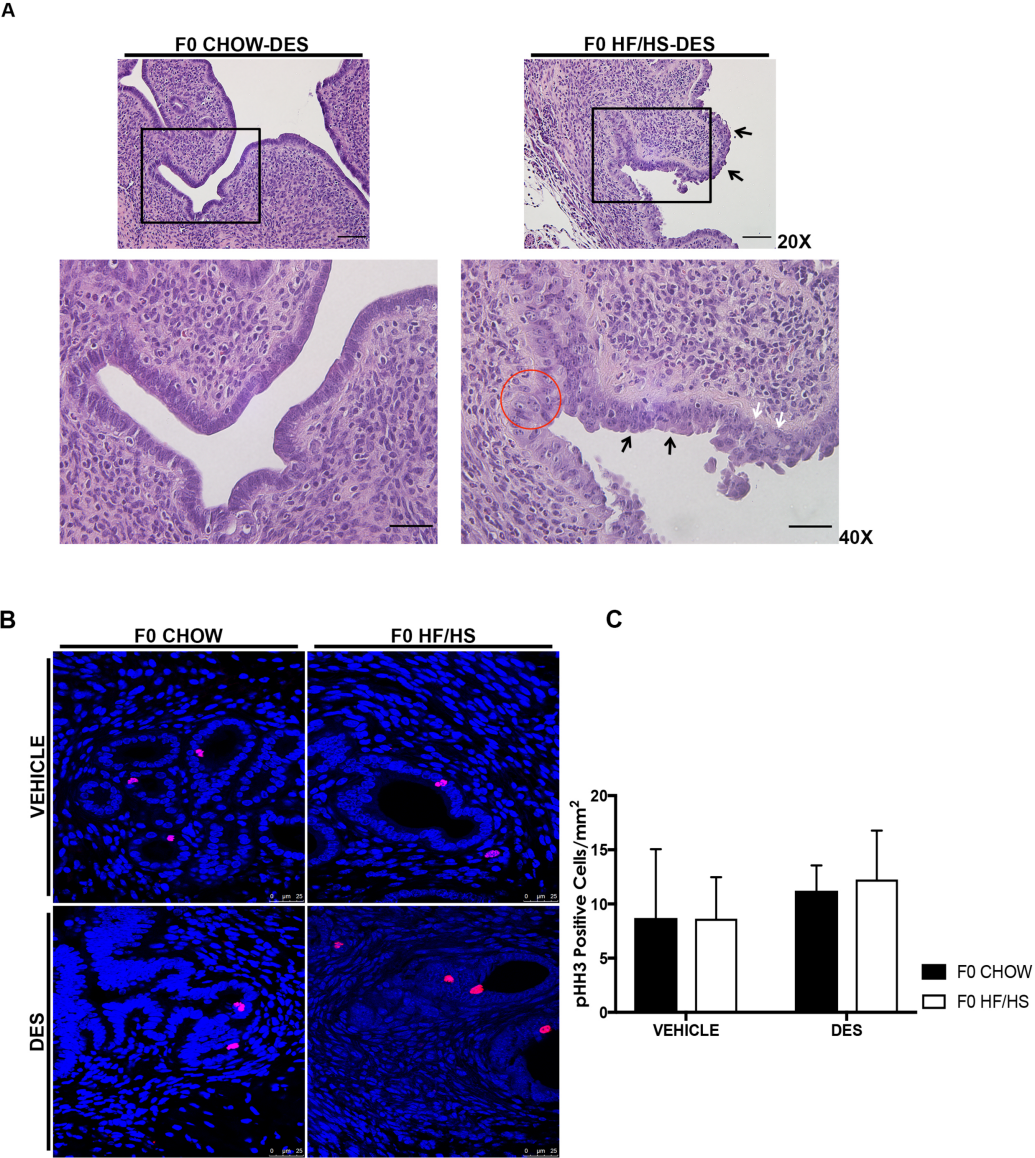
Maternal diet reduces latency of ECa in DES treated mice. Representative images of H&E slides at 20X (**upper panels**) and at 40X objective (**lower panels**), scale bars = 50 and 100 μm. (**A**). Phospho-HH3 staining of cells as a marker of proliferation (**B**). Quantification of pHH3 positive cells (**C**). Black arrow – intraluminal projections, white arrows – disorganized epithelial cells and red circle – nuclei containing 1–2 prominent nucleoli. Same letters mean there is no significant difference between conditions; different letters represent statistically significant differences at p<0.01.

We next decided to evaluate whether exposure to DES would have an additive effect on maternal obesity to cause initiation of ECa. Female offspring born from dams fed either Chow or HF/HS diet were injected with vehicle control or DES resulting in four cohorts; F0 Chow-Veh, F0 HF/HS-Veh, F0 Chow-DES, and F0 HF/HS-DES. These mice were sacrificed at 16 or 24 weeks (Fig 5A). No significant uterine lesions were observed in the F0 Chow-Veh and F0 HF/HS-Veh cohorts at either time point. At 16 weeks, the uteri of 73% (n = 15) of F0 Chow-DES and 100% (n = 13) of F0 HF/HS-DES mice had reproductive tract lesions such as reduced number of endometrial glands, simplified endometrial glands, condensed and hyalinized endometrial stroma, fibrosis and increased collagen deposition (S3 Table). As we only observed reproductive tract lesions and not pre-cancerous phenotypes at 16 weeks, we focused on 24 weeks as a time point for the rest of our experiments. At 24 weeks, the F0 Chow-DES cohort weighed significantly more than their vehicle control counterparts. The F0 HF/HS-DES cohort weighed less than the F0 Chow-DES but this was not statistically significant (S2A Fig). Uterine to body weight ratio was lower in mice injected with DES than in the vehicle-treated mice, but this was only significant between the F0 Chow-Veh and F0 Chow-DES groups (S2B Fig). The DES-treated mice also had increased areas of stromal fibrosis, hyalinization with an increased extension of the collagen deposition into the myometrium (Fig 5B, upper panels [white arrows], Table 1), resulting in extensive disruption and loss of the inner and in some instances the outer muscular layer (Fig 5B, lower panels [black arrows], Table 1). Both F0 Chow-DES (n = 12) and F0 HF/HS-DES (n = 6) mice had significantly fewer endometrial glands (5.292 and 4.24 glands per mm^2^ compared with 21.43 and 15.63 (p<0.01) in the F0 Chow-Veh and F0 HF/HS-Veh cohorts, respectively (Fig 5C and 5D). Fifty percent of the F0 HF/HS-DES mice developed features indicative of the initial stages of carcinogenesis including focal glandular hyperplasia, and atypical endometrial hyperplasia (Table 1) that was characterized by disorganized proliferation of the epithelial cells (Fig 6A, white arrows), intraluminal projections (Fig 6A, black arrows), and cells nuclei containing more than one prominent nucleoli (Fig 6A, red circle). We noted also numerous mitotic figures with H&E histology but observed no difference in the number of proliferating cells between all four groups when we quantified pHH3 positive cells (Fig 6B and 6C). None of the mice in the F0 Chow-DES cohort developed these ECa features (Fig 6A, left panels, and Table 1). In summary, exposure to maternal HF/HS diet increases offspring’s incidence of endometrial hyperplasia, an early hallmark of ECa, in mice treated with DES.

**Table 1:** Histological report of 24 week old mice exposed to either maternal Chow or HF/HS diet and injected with vehicle control (VEH) or DES.

Cohort	Mice (n)	Stromal Fibrosis	Hyalinization	Collagen deposition and extension into myometrium	Endometrial/ glandular hyperplasia
**F0 CHOW-VEH**	**12**	7	0	1	0
**F0 HF/HS-VEH**	**3**	1	0	0	0
**F0 CHOW-DES**	**12**	12	10	11	0

### Maternal diet exposure leads to an increase in phospho-Akt expression in DES treated mice

**Fig 7:**
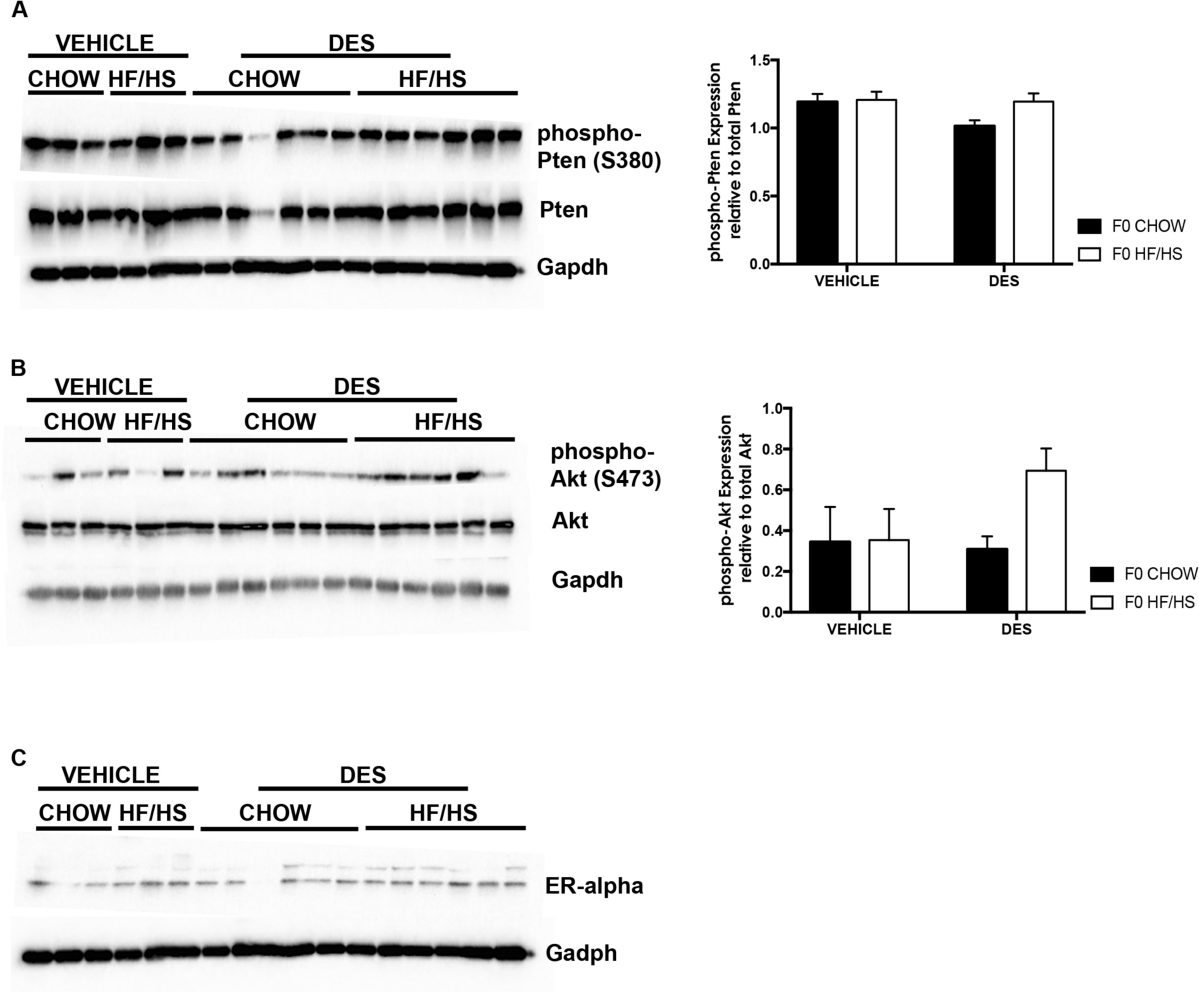
Maternal diet induces changes in phospho-Pten and phospho- Akt protein expression in DES treated mice. Immunoblot images and quantification of phospho-Pten and total Pten (**A**), phospho-Akt and total Akt (**B**), and immunoblot of Estrogen receptor α (**C**).

We next assessed expression of several proteins essential in ECa development. There was no overall change in Phospho-Pten or total Pten expression in the uteri of mice in all groups relative to expression in the F0 Chow-Veh mice data not shown. There was a 1.5 fold increase in phospho-Akt expression in mice exposed to DES and maternal HF/HS diet and no difference in expression in total Akt as expected (Fig 7B). We did not observe a change in expression of the Estrogen receptor α in mice injected with DES (Fig 7C). Our data suggest that exposure to maternal HF/HS diet induces activation of the Akt pathway in DES treated mice.

## Discussion

Obesity is a chronic disease, and is prevalent in both developed and developing countries. It has been linked to several diseases including hypertension, cardiovascular disease, type 2 diabetes, and sleep apnea, and confers an increased risk of developing several cancers including endometrial, breast, prostate, and colon cancer[43]^,^[44]. The development of cancer is complex, resulting from interactive influences from numerous factors including genetic and epigenetic alterations. Our lab has previously demonstrated that maternal obesity in mice caused by exposure to a HF/HS diet prior to conception, during pregnancy, and throughout lactation impairs early embryo and gamete development and increases male offspring’s risk of prostate hyperplasia[20, 45]. In our study, we sought to investigate the developmental origins of ECa using a maternal HF/HS diet mouse model. We further introduced a HF/HS diet to some of the F1 offspring post-weaning to assess whether this extra stimulus would further drive the risk of ECa cancer in the F1 offspring. Using this model, we show that maternal obesity induced by the HF/HS diet alone is not sufficient to increase the risk of ECa development in F1 offspring. Not only did the maternal diet by itself not induce ECa, we found that there was no additive risk of EMc as a result of feeding the HF/HS exposed F1 offspring the HF/HS diet. In previous work from our lab, we observed metabolic syndrome in 8-week-old female mice that were exposed to a HF/HS diet but fed a Chow diet[46]. Here we noted that when the F1 offspring mice were only exposed to the maternal HF/HS, they did not show any phenotypes indicative of metabolic syndrome at 39 or 72 weeks. The cohorts of mice that were fed the HF/HS diet post-weaning had significant weight gain, impaired glucose homeostasis, elevated fasting insulin and cholesterol levels, and increased percent fat mass at 72 weeks. This data suggests that at an advanced age (correlating with a peri- menopausal to menopausal age in women), maternal diet exposure has less of an impact on metabolic health, and that an individual’s diet is the driving predictor of overall metabolic health.

Obesity increases exposure to unopposed estrogen in pre- and post-menopausal women secondary to a high level of aromatization of androgens in adipose tissue[6, 46, 47]. In this mouse model, we did not observe a change in circulating estrogens (estradiol or estrone) levels with exposure to a high fat/high sugar diet even in mice that had significant weight gain and a high fat content. We also saw no change in expression of Estrogen receptor α protein in the uteri of all of cohorts of mice (data not shown).

These results could be because of two main reasons. First, the C57BL/6J strain in not susceptible to getting ECa as a result of exposure to an obesogenic environment and therefore other strains of mice may be more suitable. Walker *et al*. have previously shown that CD-1 mice get several cancers including ECa when exposed *in utero* to a high fat diet[35] but there were no follow up studies from this group on the phenotypes they observed with ECa. To our knowledge, no other group has explored exposure to maternal diet *in utero* and during early pre-pubertal development as a risk factor for ECa. There are also key differences to note between our study and those reported by Walker *et al*.: (1) We used a C57Bl/6J mouse strain, which is now the most universally used genetic background in disease models[48]. (2) We exposed the F1 offspring to maternal diet both *in utero* and during lactation while Walker *et al*. only exposed their mice *in utero*, and thus our model more closely simulates exposure of human children to their mother’s diet during gestation and breastfeeding. (3) The diet we used was composed of 59.4% fat, and 26% carbohydrates (17% sucrose) compared to 29% fat diet in the Walker study, and is more representative of fat and sugar consumption today. (4) We let the mice grow to 72 weeks after exposure to maternal diet and fed some of the mice the same HF/HS diet that had been fed to their mothers.

The second reason why we did not observe ECa in these mice may be that neither maternal nor post-weaning diet (alone or in combination) is enough to initiate ECa and that additional exposures or conditions are needed for this to occur. DES is a potent synthetic estrogen that has been previously linked to several reproductive tract lesions and cancers in mice[38]. Here, we found that 50% of mice that were exposed to a maternal diet high in fat and sugar, and then injected with DES showed early phenotypes of ECa. Additionally, these mice had an increase in activated Akt. This same activation of Akt has been described in other mice studies with DES exposed neonates and has been linked to the development of carcinoma in the vagina and endometrium[49].

Our study suggests that maternal obesity may be a determining risk factor for ECa in offspring in the presence of a secondary insult like DES. In human reproductive tract development, exposure to maternal diet does not occur in isolation. Several factors may contribute to and drive maternal influence on uterine development and ECa susceptibility. Such factors can include carcinogens such as DES, as well as somatic and hereditary mutations such as mutations in tumor suppressor genes like the PTEN gene, which is commonly found in ECa patients. In our study, we aimed to determine the impact of maternal obesity via a HF/HS diet in mice on the development of ECa in offspring. We discovered that exposure to a maternal HF/HS diet alone did not cause ECa in offspring; however, with the addition of DES, fifty percent of the exposed mice developed features indicative of carcinogenesis. Our findings suggest that maternal obesity may sensitize the endometrium to additional exposures or insults. Further studies are needed to assess genetic markers and cancer related genes in the setting of maternal diet exposure. Moreover it is not known how exposure to a HF/HS diet impacts an ECa patient’s survival with or without these other additional factors, and thus future investigation is warranted. Cancer is an accumulation of genetic changes caused by environmental, genetic, and epigenetic factors. Given the high rate of maternal obesity today, understanding its potential implications and risks of cancer in offspring is essential.

## Supporting information

S1 Fig Protein expression changes due to HF/HS diet in in the uterus of 72 week old mice. Immunoblot images and quantification of phospho-Pten and total Pten, and phospho-Akt and total Akt (**A**), and Estrogen receptor α (**B**). n = 7 mice per group.
(PDF)

S2 Fig Body weight and uterine/body weight ratio of 24 week old control and DES mice exposed to maternal Chow or HF/HS diets. (PDF)

S1 Table Histological report of 72-week-old mice exposed maternal Chow or HF/HS diet and/or fed a CHOW or HF/HS diet. (PDF)

S2 Table Histological report of 16 week old mice exposed to either maternal Chow or HF/HS diet and injected with vehicle control (VEH) or DES. (PDF)

S3 Table Primary antibodies used in western blotting ^40^ and immunofluorescence ^8^ and immunohistochemistry (IHC). (PDF)
