# Supplementary figures and images for: Maternal obesogenic diet induces endometrial hyperplasia, an early hallmark of endometrial cancer, in a diethylstilbestrol mouse model
Source: PLoS One. 2018 May 18;13(5):e0186390. doi: 10.1371/journal.pone.0186390 (PMC5959064; doi:10.1371/journal.pone.0186390)

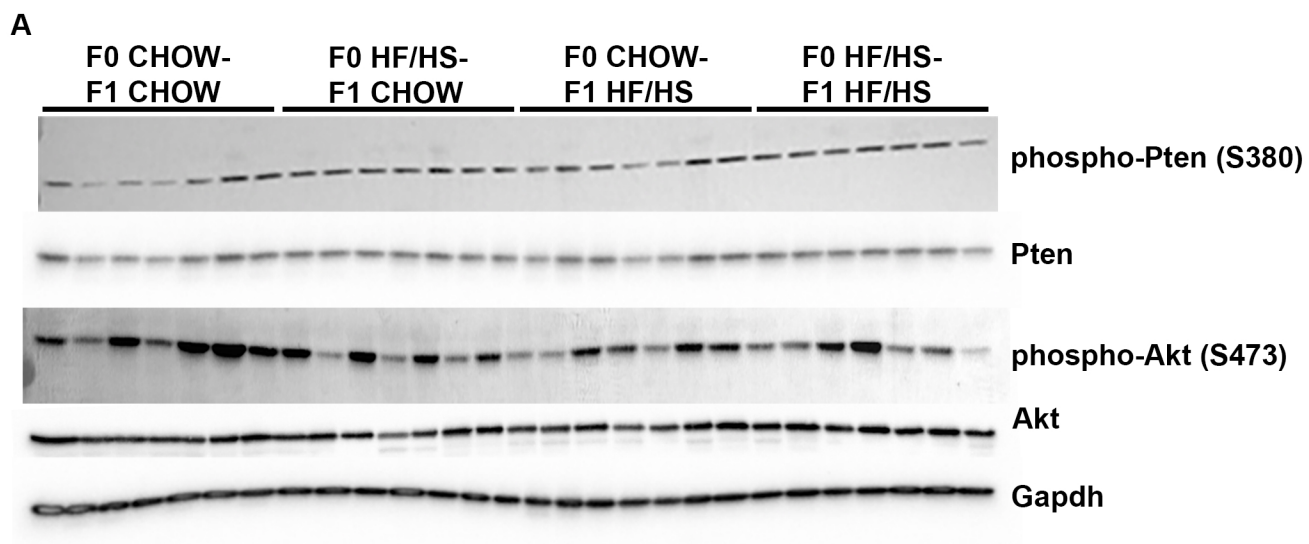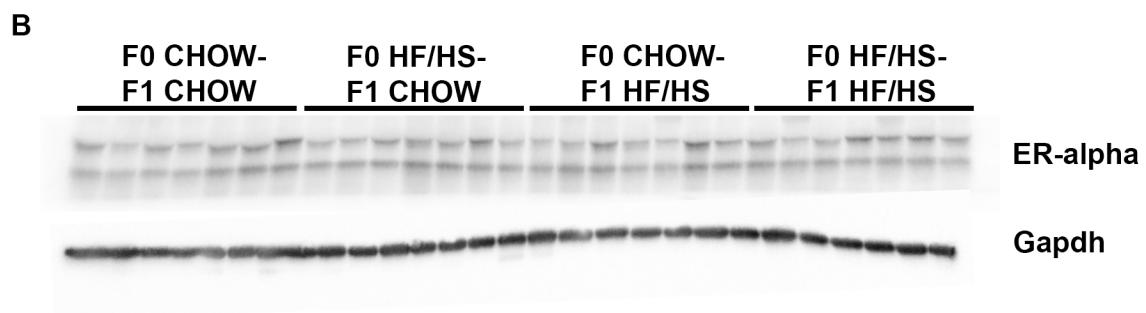

Supplementary Figure 1

Supplement: S1 Fig — Immunoblot images and quantification of phospho-Pten and total Pten, and phospho-Akt and total Akt (A), and Estrogen receptor α (B). n = 7 mice per group. (PDF) [file pone.0186390.s001.pdf]

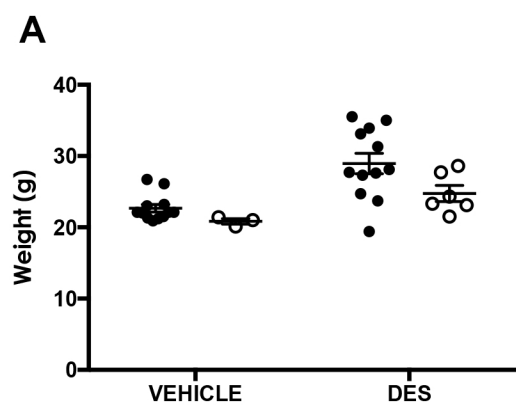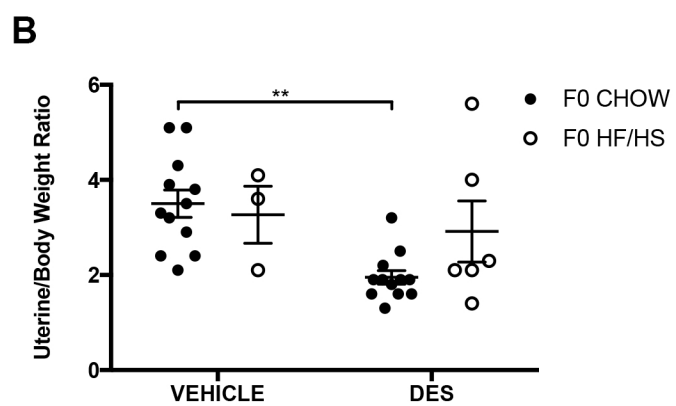

Supplementary Figure 2

Supplement: S2 Fig — (PDF) [file pone.0186390.s002.pdf]
