# Supplementary material for: Maternal obesogenic diet induces endometrial hyperplasia, an early hallmark of endometrial cancer, in a diethylstilbestrol mouse model
Source: PLoS One. 2018 May 18;13(5):e0186390. doi: 10.1371/journal.pone.0186390 (PMC5959064; doi:10.1371/journal.pone.0186390)
Supplement: S1 Table — (PDF) [file pone.0186390.s003.pdf]

Antibody	Assay	Concentration	Catalog Number	Company
ERa	WB	1:500	Ab32063	Abcam
GAPDH	WB	1:2500	2118L	Cell Signaling
Phospho-Pten (S380)	WB	1:1000	9551	Cell Signaling
Pten	WB	1:1000	9559	Cell Signaling
Phospho-Akt (S473)	WB	1:1000	4060	Cell Signaling
Akt	WB	1:1000	4691	Cell Signaling
IGF1-R	WB	1:1000	3027S	Cell Signaling
Phospho-Histone H3	IF, IHC	1:1000, 1:750	Ab5176	Abcam
αSMA	IHC	1:250	Ab5694	Abcam
CK8	IHC	1:500	Ab53280	Abcam
