# Supplementary material for: Maternal obesogenic diet induces endometrial hyperplasia, an early hallmark of endometrial cancer, in a diethylstilbestrol mouse model
Source: PLoS One. 2018 May 18;13(5):e0186390. doi: 10.1371/journal.pone.0186390 (PMC5959064; doi:10.1371/journal.pone.0186390)
Supplement: S2 Table — (PDF) [file pone.0186390.s004.pdf]

Cohort	Mice (n)	Cystic dilation	Hyalinized Stroma	Increased Neutrophils	Increased endometrial volume and complexity	Hyperplasia/ Adenocarcinoma
F0 CHOW-F1 CHOW	16	2	2	1	2	0
F0 HF/HS-F1 CHOW	20	1	0	0	2	0
F0 CHOW-F1 HF/HS	13	2	0	0	0	0
F0 HF/HS-F1 HF/HS	9	3	0	2	0	0
