# Supplementary material for: Maternal obesogenic diet induces endometrial hyperplasia, an early hallmark of endometrial cancer, in a diethylstilbestrol mouse model
Source: PLoS One. 2018 May 18;13(5):e0186390. doi: 10.1371/journal.pone.0186390 (PMC5959064; doi:10.1371/journal.pone.0186390)
Supplement: S3 Table — (PDF) [file pone.0186390.s005.pdf]

Cohort	Mice (n)	Stromal Fibrosis	Hyaliniz ation	Collagen deposition and extension into myometrium	Thinning of muscular layer	Decrease in Glands
**F0 CHOW-VEH**	**10**	2	1	1	0	4
**F0 HF/HS-VEH**	**2**	0	0	0	0	1
**F0 CHOW-DES**	**13**	11	10	10	7	10
**F0 HF/HS-DES**	**13**	13	11	13	7	11
